# Proinflammatory and Cancer-Promoting Pathobiont *Fusobacterium nucleatum* Directly Targets Colorectal Cancer Stem Cells

**DOI:** 10.3390/biom12091256

**Published:** 2022-09-07

**Authors:** Virve Cavallucci, Ivana Palucci, Marco Fidaleo, Antonella Mercuri, Letizia Masi, Valeria Emoli, Giada Bianchetti, Micol Eleonora Fiori, Gilad Bachrach, Franco Scaldaferri, Giuseppe Maulucci, Giovanni Delogu, Giovambattista Pani

**Affiliations:** 1 Department of Translational Medicine and Surgery, Università Cattolica del Sacro Cuore, Largo Francesco Vito, 1, 00168 Rome, Italy; 2 Fondazione Policlinico Universitario A. Gemelli IRCCS, L. go A. Gemelli 8, 00168 Rome, Italy; 3 Dipartimento di Scienze Biotecnologiche di Base, Cliniche Intensivologiche e Perioperatorie (Sezione di Microbiologia), Università Cattolica del Sacro Cuore, 00168 Roma, Italy; 4 Department of Biology and Biotechnology Charles Darwin, University of Rome Sapienza, 00185 Rome, Italy; 5 CNIS Research Center for Nanotechnology Applied to Engineering, Sapienza University of Rome, 00185 Rome, Italy; 6 IBD Unit, CEMAD, Medicina Interna e Gastroenterologia, Fondazione Policlinico Universitario A. Gemelli IRCCS, 00168 Roma, Italy; 7 Department of Neuroscience, Biophysics Sections, Università Cattolica del Sacro Cuore, Largo Francesco Vito, 1, 00168 Rome, Italy; 8 Department of Oncology and Molecular Medicine, Istituto Superiore di Sanità, 00161 Rome, Italy; 9 The Institute of Dental Sciences, Hadassah School of Dental Medicine, The Hebrew University, Jerusalem 91905, Israel; 10 Mater Olbia Hospital, 07026 Olbia, Italy

**Keywords:** colorectal cancer, cancer stem cells, microbiota, fusobacterium nucleatum, tumor microenvironment, tumor spheroids, carcino-embryonic antigen cell adhesion molecule-1, PTPase, bacterial adhesins

## Abstract

Intestinal bacterial communities participate in gut homeostasis and are recognized as crucial in bowel inflammation and colorectal cancer (CRC). *Fusobacterium nucleatum* (*Fn*), a pathobiont of the oral microflora, has recently emerged as a CRC-associated microbe linked to disease progression, metastasis, and a poor clinical outcome; however, the primary cellular and/or microenvironmental targets of this agent remain elusive. We report here that *Fn* directly targets putative colorectal cancer stem cells (CR-CSCs), a tumor cell subset endowed with cancer re-initiating capacity after surgery and chemotherapy. A patient-derived CSC line, highly enriched (70%) for the stem marker CD133, was expanded as tumor spheroids, dissociated, and exposed in vitro to varying amounts (range 100–500 MOI) of *Fn*. We found that *Fn* stably adheres to CSCs, likely by multiple interactions involving the tumor-associated Gal-GalNac disaccharide and the *Fn*-docking protein CEA-family cell adhesion molecule 1 (CEACAM-1), robustly expressed on CSCs. Importantly, *Fn* elicited innate immune responses in CSCs and triggered a growth factor-like, protein tyrosine phosphorylation cascade largely dependent on CEACAM-1 and culminating in the activation of p42/44 MAP kinase. Thus, the direct stimulation of CSCs by *Fn* may contribute to microbiota-driven colorectal carcinogenesis and represent a target for innovative therapies.

## 1. Introduction

Colorectal cancer (CRC) represents the third-most-commonly diagnosed cancer and the second leading cause of cancer death worldwide [1]. Although most (80%) of CRCs are diagnosed as surgically resectable, a significant fraction of them (25–30%) will relapse in 3–5 years of surgery, notwithstanding the resort to adjuvant chemotherapy [2], in a fashion that is difficult to predict based on current biomolecular and clinical knowledge. A better understanding of the biological causes and molecular interactions leading to the onset and clinical progression of this life-threatening disease is, therefore, imperative.

While somatic DNA mutations fuel cancer initiation and evolution in CRC and other malignancies, additional environmental factors, including chronic inflammation and intestinal microbes, are increasingly recognized as causative or complicit in colorectal carcinogenesis [3]. In particular, converging lines of investigation have identified bacterial pathogens that are not part of the normal intestinal microbiota, such as *Fusobacterium nucleatum* (*Fn*) [4] and *Streptococcus gallolyticus* (*Sg*) [5], as potential etiologic factors in CRC based on the over-representation of DNA sequences or even cultivable microorganisms in malignant tissues compared with normal colon specimens. Notably, *Fn* also promotes the chemoresistance of colon cancer cells, and *Fn* presence predicts lower patient survival [6]. CRC-associated bacteria promote cancer by perturbing the equilibrium among different microbiota components and between bacterial populations and inflammatory and immune cells so as to trigger a vicious dysbiosis–inflammation circle [7]. Additionally, specific pathogens directly attack and invade epithelial cells, hijacking signaling components and cascades that control cell proliferation and normal mucosal repair. More specifically, *Fn* modulates E-cadherin/β-catenin signaling via its FadA (Fusobacterium adhesin A) so as to promote oncogenic and proinflammatory responses in CRC cells [8]; the same pathogen uses the lectin Fap2 to bind the tumor-cell-expressed disaccharide Gal-GalNac [9], and host cell binding and invasion leads to the secretion of the chemokines IL-8 and CXCL1 and enhanced CRC cell migration [10]. Moreover, on the stromal tumor side, *Fn* binding to the T lymphocyte inhibitory receptors TIGIT and CEACAM-1 (via Fap-2 and the trimeric autotransporter adhesin CbpF, respectively [11]) suppresses antitumor immunity, thereby indirectly promoting malignant growth [12,13]. Importantly, *Fn* has been detected and cultivated from distant CRC metastatic lesions [4], suggesting that either this microbe localizes to disseminated cancer colonies through the hematogenous route, or can stably persist within colonic metastasis-initiating cells.

Over the last few years, intense research has highlighted the presence, in several solid malignancies, including CRC, of a unique subset of “stem-like” cells endowed with tumor-initiating capacity that are arguably responsible for cancer metastatic spreading and local recurrence after surgery. These colorectal cancer stem cells (CR-CSCs) may truly descend from intestinal stem cells undergoing oncogenic transformation, or rather reflect the de-differentiation of more mature or even fully differentiated enterocytes; either way, they can be identified by the expression of molecular markers (CD133, EpCAM, CD44v6, LGR5, ALDH1, and DCLK1) and the activation of intracellular signaling pathways (Wnt-APC/β-catenin, Notch, TGF-β/BMP) (see [14] for a comprehensive literature review) functionally related to self-renewal activity and pluripotency.

While several studies have investigated the molecular determinants and downstream functional consequences of the interaction between oncomicrobes and colon cancer epithelial and stromal/immune cells, little information is available on whether such interactions also or even preferentially involve CSC. It is, however, known that colorectal-tumor-initiating cells exploit autocrine cytokine-triggered circuitries to resist chemotherapy-induced cell death [15], and that normal intestinal stem cells express innate immune receptors that mediate protection from oxidative damage and ROS cytotoxicity in response to bacterial components [16]. Additionally, *Fn* induces CSC-like traits in cultured CRC lines via epithelial–mesenchymal transition (EMT) [17], and the *Fn*-derived metabolite formate enhances the stemness and self-renewal capacity of patient-derived colorectal tumor organoids [18].

In the present work, we set out to address the microbe–CSC interaction by exposing CSC-enriched primary spheroidal cultures of colorectal tumors to *Fn* in vitro. Our results demonstrate that *Fn* avidly binds to colonsphere-derived cells and triggers intracellular proinflammatory and oncogenic cascades superimposable to those previously described in mature CRC cells. Additionally, we highlight the role of the cellular CEACAM-1 and its associated tyrosine phosphatase SHP-2/PTPN11 in mediating early phosphorylation responses downstream of cell–pathogen interactions. These findings provide original information on the role of *Fn* in CRC and suggest a novel paradigm of bacterial carcinogenesis centered on the direct bacterial targeting of cancer-initiating cells.

## 2. Materials and Methods

**Cell lines**. The primary spheroidal colon cancer cultures CSC-P and SA-22 used in the present study were initially derived at Istituto Superiore di Sanità, Rome, Italy, and made available to GBP under a Material Transfer Agreement. The procedure of isolation and characterization is described in detail in ref. [19]. Briefly, surgical specimens of primary CRCs were cut into small pieces, digested in Collagenase II + DNAse for 1 h at 37 °C, and resuspended in a serum-free defined growth medium containing 10 ng/mL human bFGF and 20 ng/mL human EGF. CSC-enriched spheroids developing within 2–4 weeks from the primary seeding were expanded and further characterized for their mutational profile and in vivo tumorigenicity. CSCs were routinely maintained in CSC medium (Neurobasal-A or Advanced DMEM/F12, supplemented with Vitamin A-free B27 (Life Technologies, Carlsbad, CA, USA), 10 mM of nicotinamide (Sigma, St. Louis, MO, USA), 1 μM of RhoK inhibitor Y-27632 (Tocris Biosciences, Bristol, UK; cat. #1254), 6 g/L of glucose, 2 μg/mL of Heparin (StemCell Technology, Vancouver, BC, Canada; cat. #07980), 10 ng/mL of bFGF, and 20 ng/mL of hEGF (Peprotech, Thermo Fisher Scientific, Waltham, MA, USA) and passaged weekly. Spheroid aggregates were dissociated by gentle trypsinization; cell suspensions were passed through a 70 μm-pore-size strainer (FlowMi^®^, SP Bel-Art, Wayne, NJ, USA; cat. #136800070) and re-seeded at 1.5 × 10^5^ cells/mL in 25 cm^2^ ultralow-attachment tissue culture flasks. In order to induce intestinal differentiation, the cells were dissociated and cultivated for 7–10 days in regular (attachment-permissive) tissue culture plates in CSC medium supplemented with 2% FBS and 10 mM sodium butyrate (Sigma Aldrich).

The colorectal carcinoma cell lines CaCo-2 (cat. HTB-37™) and HT-29 (cat. HTB-38™) were obtained from the American Type Culture Collection (ATCC) and routinely grown in Dulbecco’s Modified Eagle’s Medium (DMEM) supplemented with 10% FBS (*v*/*v*) and antibiotics. Both lines were periodically checked for mycoplasma infection.

**Bacterial strains**. *Fusobacterium nucleatum subsp. nucleatum* Knorr (25586™) was obtained from ATCC and maintained in anaerobiosis according to the accompanying instructions. The *Streptococcus gallolyticus* (*Sg*) used in the present study was a clinical strain isolated from a patient hospitalized at IRCCS Fondazione Policlinico A. Gemelli—Università Cattolica del Sacro Cuore (Rome). *Sg* was grown at 37 °C in brain–heart infusion (BHI) broth with shaking or on BHI agar (Difco Laboratories, Sparks, MD, USA, under aerobic conditions) [5]. The *Escherichia coli* strain C43 expressing the fusobacterial adhesin CbpF (variant 1) and the relative control strain are described in ref. [20]. Recombinant *E. coli* strains were grown in LB broth (Difco) or on LB agar plates (Difco) containing 100 μg/mL of ampicillin at 37 °C under aerobic conditions. CbpF1 expression was induced with 0.4 mM of isopropyl-b-d-thio-galactoside (IPTG, Sigma) at 22 °C overnight.

**Antibodies.** The mouse monoclonal antibodies anti-CEACAM-1 (E1, cat. #sc-166453), anti-GFP (B2, cat. #sc-9996), and anti-p-(Ser 32) IkB-α (clone B9, cat. #sc-8404), as well as the polyclonal anti-SHP-2 and anti-SHP-1 rabbit antibodies (C-18, cat. #sc-280 and C19, cat. #sc-287, respectively), were obtained from Santa Cruz Biotechnology. The mouse/rat monoclonal antibodies anti-β-actin (8H10D10, cat. #3700), anti-E-Cadherin (24E10, cat. #3195), anti-GSK3β (27C10, cat. #9315), and anti-p(Ser 9) GSK3β (5B3, cat. #9323), and the polyclonal rabbit antibodies anti-p(Thr202-Tyr204) p44/42 MAPK (cat. #9101) and anti-p(Tyr 542) SHP-2 (cat- #3751) were purchased from Cell Signaling Technology. The rabbit polyclonal antiserum anti-p42/44 MAP Kinase ½ (Erk1/2) was obtained from Millipore/Merck (cat. #06-182). The PE-conjugated anti-hCD133/1 (AC133, cat. #130-113-670) and anti-hCEACAM1/CD66a (282640, cat. #FAB2244P), used for flow cytometry, were from Miltenyi Biotec and R&D Systems, respectively.

**Plasmids.** The double-color lentiviral Wnt-reporter TOP-GFPmC was a gift from Ramesh Shivdasani (Addgene plasmid #35491; http://n2t.net/addgene:35491 (accessed on 11 July 2022); RRID:Addgene_35491) [21]. This third-generation lentiviral construct encodes eGFP under the transcriptional control of 7× TCF/LEF promoter elements, while constitutive PGK-driven mCherry fused to H2B marks successfully transduces cells.

The pre-designed shRNA lentiviral construct directed against human CEACAM-1 (cat. #SHCLNG clone TRCN0000377692) and the MISSION^®^ pLKO.1-puro non-target shRNA control vector (cat. #SHC016) were purchased from Sigma/Merck. The NF-kB-responsive *Firefly* luciferase reporter construct and the CMV-driven *Renilla* internal control (Cignal Reporter Assay, cat. CCS-013L) were from QIAGEN. The IPTG-inducible plasmid encoding the two SHP-2 SH2 domains (N- and C-terminal) fused with GST (pGEX SHP-2(NC)-SH2) was a gift from Bruce Mayer (Addgene plasmid #46499; http://n2t.net/addgene:46499 (accessed on 11 July 2022); RRID:Addgene_46499) [22]. The plasmid originally provided in the DH5α *E. coli* strain was amplified, purified, and transformed into the protease-deficient BL21 strain to maximize GST-fusion protein recovery.

**CSC infection and Luciferase reporter assays**. Lentiviral supernatants were produced according to Tiscornia et al. [23], with minor changes; the plasmid mixture containing the transfer vector, pMDL, pRev, and pVSVG, was introduced to HEK-293T cells by calcium phosphate precipitation. Supernatants from the second and third days post-transfection were pooled and concentrated 100 times by ultracentrifugation (72,000 g for 2 h at 20 °C). Pooled supernatants from one or two 10 cm Petri dishes (4–8 × 10^6^ packaging cells) were used to infect 2 × 10^5^ CSC cells in 2 mL of complete CSC medium. To increase the infection efficiency, cells were co-centrifugated with lentiviral particles at RT for 2.5 h at 2500 g (“spinoculation”) in presence of 8 μg/mL of polybrene (Sigma-Aldrich/Merck, Darmstadt, Germany).

For the transfection of *luc* reporter plasmids, 2.5 × 10^5^ cells from freshly dissociated spheroids were seeded in 0.5 mL of complete CSC medium without Heparin (found to interfere with transfection) and left to recover for 8 h. Plasmid DNA (1 μg of *Firefly* reporter and 25 ng of CMV-*Renilla* internal control) was transfected using the Lipofectamine 3000 reagent (Life Technologies, Carlsbad, CA, USA) in 24-well ultralow-attachment cell culture clusters. After overnight incubation, stimuli were applied as needed. Following an additional 24 h, the normalized reporter activity (*Firefly/Renilla*) was measured by luminometry using a dual luciferase assay kit (Promega, cat. E1910) according to the manufacturer’s instructions.

**Flow Cytometry.** For FC analysis, spheroid cultures were dissociated with trypsin, and cell suspensions were filtered through a 70 μm cell strainer. For surface staining, cells (5 × 10^5^ in 75 μL) were incubated in PFA buffer (PBS + 1% FBS + 0.05% Azide) with 0.5–1 μg of primary antibody for 60 min on ice, followed by two washes in cold PFA. Labeling with a secondary reagent, if necessary, was performed sequentially following an identical procedure. For cell staining with FITC-labeled peanut lectin (PNA, Sigma/Merck, cat. #L7381), 2.5 × 10^5^ cells were incubated in 100 μL PFA buffer with 2 μg/mL lectin for 30 min on ice.

Samples were analyzed with either a three-laser, 12-fluorescence Cytoflex cytometer (Beckman Coulter, Brea, CA, USA) or with a single-laser (488 nm), 3-fluorescence MCL-XL Epics (Coulter) instrument. Dead cells were excluded by staining with propidium iodide or based on their position in the forward-scatter/side-scatter plot.

**Detection of Nitric Oxide by DAF-FM**. Dissociated CSC-P cells (2.5 × 10^5^) were seeded in ultralow-attachment 24-well clusters in 500 μL of complete CSC medium without antibiotics and incubated for 24 h with 200 or 500 MOI *Fn.* DAF-FM diacetate (Thermo-Fisher, cat. #D23844) was added at 10 μM for the last 60 min of incubation. Cells were then washed in cold PBS, re-dissociated with trypsin, and immediately analyzed by flow cytometry.

**Bacterial pull-down assay** (*Bactoprecipitation*). In order to isolate CSC proteins potentially involved in cell interaction with bacteria, 0.5–1 × 10^6^ cells were lysed in 100 μL of PBS containing 1% Triton X-100 and protease inhibitors, incubated on ice for 10 min, and spun down at 14,000 rpm, 4 °C to remove unlysed cells, nuclei, and cell debris. Next, the supernatant was mixed with 900 μL of PBS containing 10^9^ bacterial cells (final Triton X-100 concentration 0.1%) and incubated at 4 °C for 45 min on a rotating wheel. Bacteria were then centrifuged at 3000× *g* for 10 min, washed twice in cold PBS, and finally resuspended in 60 μL of 1× SDS Laemmli sample buffer (50 mM Tris-HCl pH 6.8, 5% β-mercaptoethanol, 10% glycerol, 1% SDS, and 1.5 mM bromo-phenol blue), briefly sonicated, and boiled for 5 min to elute bacteria-adsorbed cellular proteins. After a brief centrifugation step, the supernatants were used for regular Western blot analysis.

***Fn* fluorescent labeling and CSC–bacteria binding assay**. *Fn* and other bacterial strains were fluorescently labeled using the Bac-Light™ Red (Thermo Fisher, Waltham, MA, USA; cat. #B35001) or the BactoView™ Live Green (Biotium, Fremont, CA, USA; cat. #40102) bacterial stains according to the manufacturer’s indications. After two washes in PBS to remove the unbound dye, bacteria were mixed with CSC in a 100:1 ratio and incubated at 37 °C for 30 min in 500 μL of RPMI, with occasional agitation. Samples were then briefly centrifuged (14,000 rpm for 15 s) to separate cells from unbound bacteria, washed once in RPMI, and analyzed by flow cytometry. Labeled bacteria were also separately analyzed to identify and gate out the corresponding population on the FS-SS plot. Comparable labeling intensities among different strains (i.e., *Fn* vs. *Sg*) were also verified.

**Confocal microscopy imaging**. The binding of Red-*Fn* after 30 min of CSC co-incubation with fluorescent bacteria was qualitatively assessed by confocal microscopy imaging [24,25]. Images were acquired with a Nikon A1-MP inverted confocal microscope equipped with an on-stage incubator (OKOLAB), which kept a constant temperature of 37 °C and 5% CO_2_. Fluorescence emission, excited with a single-photon laser (excitation wavelength: 561 nm), was collected in the wavelength range of 570–620 nm using a 60× oil-immersion objective (1.4 NA). Brightfield images, collected along with fluorescent images, were used to highlight the distribution of red-stained bacteria.

**Cell stimulation.** CSCs from dissociated spheroids were counted with a hemocytometer, spun down at 14,000 rpm for 20 s, and resuspended in RPMI without additives at 0.5–1 × 10^7^ cells/mL in 100 μL (short-term stimulation) or 1 × 10^6^/mL in 500 μL (16–24 h stimulation). Bacterial liquid cultures were spun down at 3000 g (3900 rpm) for 10 min, washed once in PBS, and quantified by spectrophotometry at 660 nm (1 OD = 10^9^ cells/mL) [26]. The desired bacterial MOI were resuspended in 100 μL of RPMI, briefly (5 s) sonicated to dissolve gross aggregates, and mixed with CSCs (final stimulation volumes of 200 μL and 600 μL, respectively). At the end of the incubation, the cells were quickly pelleted in a benchtop centrifuge (14,000 rpm × 20 s) and flash-frozen for further processing or directly lysed in 80 μL of 1× SDS Laemmli sample buffer. For overnight incubation, Gentamycin (100 μg/mL) was added to the culture after 3 h of stimulation to prevent bacterial overgrowth in the medium.

**Western blotting.** Protein samples in Laemmli buffer were heated at 95 °C for 5 min, subjected to SDS-PAGE, and electroblotted onto a nitrocellulose membrane. Immunocomplexes were visualized by enhanced chemiluminescence (Westernbright™ ECL, Advansta, San Jose, CA, USA, cat. #K-12045) with the Alliance Q9^®^ advanced chemiluminescence imager (Uvitec, Cambridge, UK). Digital images were quantified using the Image J software (Analize→Gels).

**Cell lysis and immunoprecipitation.** For co-immunoprecipitation studies, cell pellets (3–5 × 10^6^ cells) were lysed in 1 mL of cold lysis buffer (50 mM Tris-HCl pH 8.0; 150 mM NaCl; 5 mM EDTA; and 0.05% Na+ Azide) containing 1% (*v*/*v*) Triton-X100, and protease and phosphatase inhibitors. After 15 min of incubation on ice, the tubes were spun down (14,000 rpm for 10 min at 4 °C) to remove cell debris and unlysed nuclei, and the supernatants were precleared with empty Protein A/G sepharose beads (100 μL of a 10% *v*/*v* slurry) for 1 h at 4 °C on a rocking plate. After centrifugation, 1/20 of the supernatant was kept for Western blot analysis (input) and 19/20 was incubated with 1 μg of antibody (anti-CEACAM-1 or anti-SHP-2) and 100 μL of protein A/G slurry for 16 h in rotation at 4 °C. Sepharose-bound immunocomplexes were collected by centrifugation (14,000 rpm × 30 s), washed 4–5 times in lysis buffer with inhibitors, eluted in Laemmli buffer and analyzed by Western blotting.

**GST-pull down assay**. To obtain the immobilized GST-2SH2-SHP2 fusion protein, the encoding *pGEX* plasmid (Addgene #46499) was transformed into the low-protease *E. coli* strain BL21; overnight bacterial cultures were diluted 1:10, incubated until OD > 0.6, and induced for 3 h with 1 mM isopropyl β-D-1-thiogalactopyranoside (IPTG). Pellets were lysed in PBS/Triton-X100 1%/PMSF, and the GST fusion protein was affinity-purified with Glutathione-Sepharose (10 µL packed resin/mL lysate, 30 min at RT) in batch, extensively washed, aliquoted, and frozen. For the pull-down assay, Sepharose-bound GST-2(SH2)-SHP2 (the equivalent of 1 mL of bacterial culture) was incubated with CSC lysates, obtained as for a conventional immunoprecipitation, for 2.5 h at 4 °C in rotation. The following washing and elution steps were conducted as for immunoprecipitation. After protein blotting, equal amounts of the GST fusion proteins throughout the samples were verified by membrane-reversible Ponceau S staining.

**Multiplex cytokine screening of CSC supernatants.** Cells (2 × 10^6^ in 2 mL CSC medium) were stimulated for 24 h with 100 MOI *Fn* or left untreated. Supernatants were overlaid on twin membranes of a semi-quantitative, sandwich-based, antibody array kit (Cytokine Array C3, Ray Biotech, Peachtree Corners, GA, USA, cat. #AAH-CYT-3) and incubated for 16 h at 4 °C on a rocking plate. Subsequent steps for chemiluminescent immunocomplex detection were conducted according to the manufacturer’s instructions.

**Quantitative Real-Time PCR.** For RNA extraction, undissociated CSC spheroids or plastic-adherent (differentiated CSCs or HT-29 and CaCo_2_ cultures) cell clusters were washed once in PBS and processed using a Direct-zol RNA Miniprep kit (Zymo Research, Irvine, CA, USA, R2052) according to the manufacturer’s instructions. The amount and purity of RNA were determined by NanoDrop™ (Thermo Fisher Scientific). SensiFAST™ cDNA Synthesis Kit (Meridian Bioscience, Cincinnati, OH, USA) cDNA was used for RNA retro-transcription. Real-time PCR was performed using a SensiFAST™ SYBR^®^ No-ROX Kit (Meridian Bioscience) with a CFX96 qPCR Instrument (Bio-Rad, Hercules, CA, USA). Primer sets for human NANOG (amplicon size 129 bp), human OCT4/POU5F1 (amplicon size 154 bp), and the human housekeeping gene LDHA (a.s. 130 bp) were from the Human Stem Cell Pluripotency Detection qPCR Kit (ScienCell, Carlsbad, CA, USA, Catalog #0853). The reaction conditions were as per the manufacturer’s recommendations.

To detect *Fn* DNA in CSC-P cultures, genomic DNA was obtained from cell pellets (10^6^ cells) following a standard procedure. A 244 bp fragment from the *Fn* (subspecies *nucleatum*, MT482608.1) 16S ribosomal RNA gene was amplified using the following primer pair: F: AAAGCGCGTCTAGGTGGTTA and R: GTTTACGGCGTGGACTACCA.

**Cell viability assay.** CSC-P cells were seeded in complete medium at 2.5 × 10^4^/100μL in 96 flat-bottom well plates and treated for 4 days with the alkylating agent Oxaliplatin (Selleckchem, Houston, TX, USA, cat. #S1224) at 250–7.5 μM or left untreated. A total of 250 heath-killed MOI (H-K, 60 °C for 40 min.) were added immediately after seeding where needed. Cell survival was measured by the CellTiter-Glo^®^ Luminescent Cell Viability Assay (PROMEGA, Madison, WI, USA; cat. #G7571) according to the manufacturer’s instructions. In each sample group (control and H-K *Fn*), the average RLU reading (background-subtracted) of the untreated wells (No Oxa) was assumed as 100% survival, and cell viability throughout the treatments was calculated as (RLU well/average RLU of untreated wells) × 100. RLU readings from wells containing H-K *Fn* without CSC-P cells were not different from the background.

**Statistical analysis.** Differences between two sample means were evaluated by a two-tailed Student’s *t*-test for independent or correlated samples, where appropriate. One- or two-way ANOVA followed by Tukey Honest Significant Difference post hoc analysis were used for multiple comparisons. Experimental measurements from independent experiments (i.e., control vs. *Fn*) were grouped and analyzed pairwise by the Wilcoxon signed rank test, or by ANOVA for correlated samples if k > 2. Where data were reported as the fold induction (compared with untreated control), relevant statistical tests were performed for row values or stimulation indexes (treated/untreated). The single-sample Student’s *t*-test (two-tailed) was used to compare the mean fold induction value with the null hypothesis = 1 (no effect). The threshold for statistical significance was set at *p* < 0.05 (two-tailed). Calculations were performed on the online Vassar Stats platform (http://vassarstats.net/index.html, accessed between 1 March–1 May 2022).

## 3. Results

### 3.1. Oncobacterium Fusobacterium Nucleatum Directly Targets Human Stem-Like Colorectal Cancer Cells

**Figure 1:**
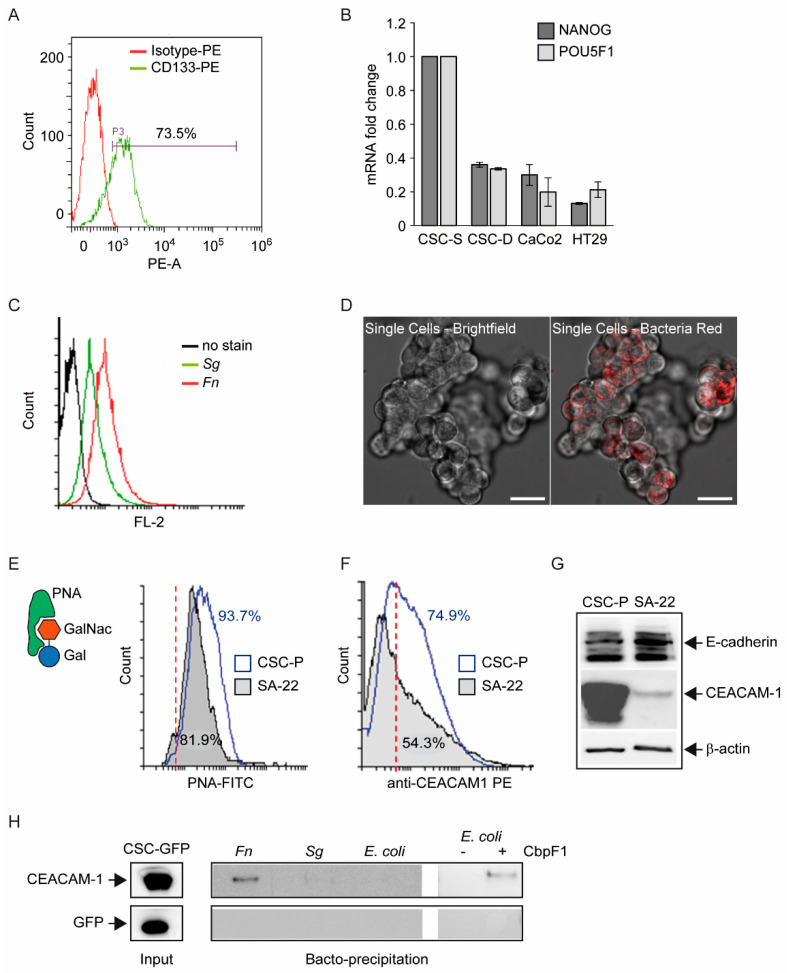
*Direct interaction between Fn and spheroidal colon cancer stem cells*. (**A**) Flow cytometry analysis for the stem cell marker CD133 in spheroidal CSC-P cells. The percentage of the CD133+ population is indicated. (**B**) Relative expression level of *NANOG* and *POU5F1/OCT4* mRNA in CRC lines CaCo_2_ and HT29 compared with primary colonsphere cells (CSC) grown in proliferative (S) or differentiative (D) medium. Values are the mean ± SD of qPCR experimental duplicates. (**C**) Flow cytometry plot of CSC-associated fluorescence following incubation with 100 MOI of red-labeled bacteria. Black trace: baseline; *Fn*: *Fusobacterium nucleatum*; *Sg*: *Streptococcus gallolyticus*. (**D**) Representative confocal images of red-fluorescent *Fn* adhering to freshly dissociated CSCs. Left: phase-contrast image; right: transmittance/fluorescence overlay. (**E**) Flow cytometry plot of FITC-PNA (peanut agglutinin)-stained CSC-P and SA-22 cells. Numbers indicate the % of cells exposing the Gal-GalNac sugar moiety, recognized by PNA. The red dashed line marks the positivity threshold (autofluorescence). (**F**) Single-parameter flow cytometry histogram for surface CEACAM-1 expression in spheroidal cultures of CSC-P and SA-22 cells. Numbers denote the % of cells above the isotype control (vertical dashed line) threshold. (**G**) CEACAM-1 and E-cadherin immunodetection in protein homogenates from CSC-P and SA-22 cells. β-actin serves as a control for equal sample loading. Relevant bands are indicated by arrows. (**H**) Immunoblot analysis of bacteria-adsorbed proteins after 30 min of incubation with homogenates from GFP-transduced CSCs. The input lysate is shown as a positive control for staining. The different bacterial strains used as baits are indicated in italics. Picture representative of 2–3 independent experiments with comparable results.

Several published studies have reported cancer-related effects of *Fn* infection on commercial colorectal tumor cell lines (HCT-116, HT-29, and CaCo_2_ among the others), grown in 2D culture [9,10,24] Under those conditions most cells display a mature phenotype, although stem-like elements can still be isolated and expanded in serum-free media [27]. Of note, *Fn* has been shown to increase the expression of stem cell markers in HCT-116 cells [17]. In order to address whether *Fn* can also target immature, stem-like CRC cells, we took advantage of a patient-derived CSC line (CSC-P) cultivated in defined serum-free medium and previously characterized for its tumorigenicity in vivo [19]. CSC-P cells grow in tridimensional spheroids and express the surface stem cell marker CD133 in a high percentage (>70%) (Figure 1A). Moreover, qPCR analysis revealed that messenger RNA for the pluripotency factors *Nanog* and *POU5F1/Oct4* was 4–5-fold more abundant in these cells than in the broadly used CRC lines HT29 and CaCo_2_ (Figure 1B). Notably, both mRNAs were markedly downregulated after ten days of growth on an adhesion-permissive surface in the presence of FBS and sodium butyrate, an established protocol for CSC differentiation (Figure 1B) [19]. These preliminary studies thus confirmed the well-established notion that most spheroid-forming cells have cancer stem cell features.

To begin investigating the potential interaction between *Fn* and CSCs, cells from dissociated spheroids were incubated with fluorescently labeled live bacteria (100:1 MOI, i.e., bacteria-to-cell ratio) and cell-bound fluorescence was quantified by flow cytometry. Near-100% spheroid-derived cells appeared to bind red-labeled *Fn* after 30 min of incubation at room temperature (Figure 1C,D). Although less avidly, *Sg* also adhered to CSCs (Figure 1C). *Fn*-labeled cells remained fluorescent for several days after labeling, and bacterial DNA was still detected by qPCR after 10 weeks and as many cell passages (Supplementary Figure S1A,B).

A number of *Fn*-docking molecules on target cells have been identified, including the colon-cancer-associated carbohydrate moiety Gal-GalNac (via bacterial Fap-2) [9], E-cadherin (through the *Fn* adhesin FadA) [8], and the carcinoembryonic antigen-related cell adhesion molecule family member 1 (CEACAM-1), via the trimeric autotransporter adhesin CbpF [11]. We confirmed the presence of Gal-GalNac on the surface of CSCs by cell incubation with 2 μg/mL of FITC-labeled peanut lectin (PNA) followed by flow cytometry (Figure 1E). Likewise, Western blot analysis of cell homogenates demonstrated robust expression of both E-Cadherin and CEACAM-1 (Figure 1G), and 70% of spheroid-forming cells were found to be positive for the surface expression of CEACAM-1 by FC analysis, although with a large distribution width across the cell population (Figure 1F). Varying levels of CEACAM-1 expression were also observed among different patient-derived CSC lines, ranging from high (as in CSC-P) to barely detectable in SA-22 (Figure 1F,G).

To gain further insight into the molecular interactions between our stem cell population and *Fn*, bacteria were incubated with whole homogenates from GFP-expressing CSCs, washed, and subjected to Western blot analysis for putative surface-adsorbed mammalian docking proteins (“bactoprecipitation”). By this procedure, we readily detected CEACAM-1, but not E-cadherin (Supplementary Figure S2) or GFP (negative control) in the bacterial pellet, suggesting that the *Fn*-CEACAM-1 is not only specific, but also sufficiently strong to survive our harsh experimental conditions (Figure 1H). Importantly, CEACAM-1 was not precipitated by *E. coli* (DH5α) or *Sg*, but bound to a recombinant *E. coli* strain expressing the *Fn* adhesin CbpF [20] (Figure 1H). Taken together, these observations underscore the relevance of CEACAM-1, possibly via bacterial CbpF, as a primary molecular target for *Fn* on colorectal CSCs.

### 3.2. Fn Evokes Proinflammatory and Oncogenic Responses in CSCs

**Figure 2:**
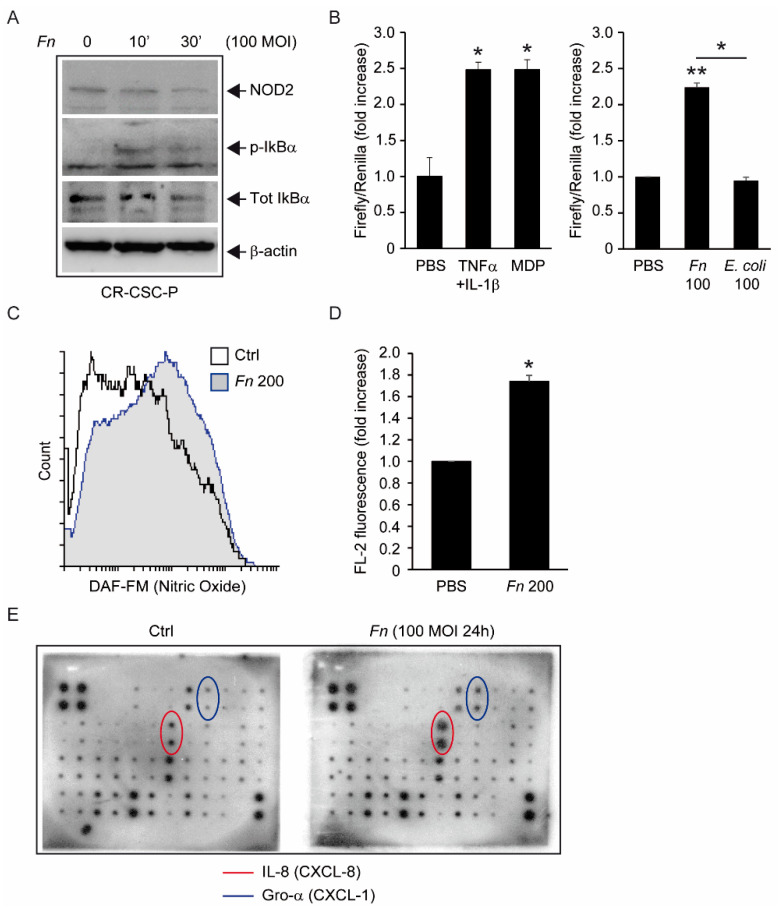
*Proinflammatory signaling of Fn in CSC.* (**A**) Immunoblot analysis of CSC-P homogenates following short (10′ and 30′) exposure to 100 MOI *Fn*. Bands corresponding to the bacterial sensor NOD2, the phosphorylated (Ser 32) form of the NF-kB inhibitor IkB, total IkB, and β-actin (loading control) are highlighted by arrows. Picture representative of at least three independent experiments. (**B**) NF-kB luciferase reporter assay in CSC-P cells transfected with a 3kB-luc reporter and exposed to whole bacteria (*Fn* or *E. coli* DH5alpha, 100 MOI each), 10 μg/mL of MDP, or TNFα + IL1β (10 ng/mL each, positive control) for 24 h. Values are the mean ± S.E.M. luminescence of duplicate/triplicate samples, normalized to the mean of untreated controls (PBS). * *p* < 0.05 compared with PBS (ANOVA/Tukey HSD). ** *p* < 0.005, single-sample *t*-test, two-tailed. * between columns: *p* < 0.05, unpaired *t*-test. (**C**) Representative flow cytometry histogram of CSC-P cells loaded with the nitric oxide-sensitive dye DAF-FM. Fluorescence distribution of unstimulated (empty histogram) and *Fn*-treated (200 MOI, 24 h, gray-filled histogram) cells are overlayed. (**D**) Mean cell fluorescence as in C, averaged from two independent experiments. Values are the mean ± S.E.M. of fluorescence intensities in the FL-2 channel, normalized to the value of the untreated (PBS) sample. * *p* < 0.05 by single-sample Student’s *t*-test (two-tailed). (**E**) Protein array hybridization comparing 42 inflammatory cytokines in the supernatants of CSC-P cells exposed to 100 MOI *Fn*, or left untreated. Each cytokine was spotted in duplicate. Spots corresponding to IL-8 (CXCL-8, red) and Gro-α (CXCL-1, blue) are circled. Picture representative of two independent hybridizations.

Having demonstrated a direct interaction of *Fn* with spheroid-forming cells, we moved on to characterize bacteria-induced downstream biological responses along the lines highlighted by previous studies on CRC cell lines. While *Fn* has been convincingly shown to trigger Toll-like receptor (TLR)-dependent inflammatory cascades in CRC cells [6,28], knowledge of innate immune signaling in normal intestinal stem cells (ISC) is still in its infancy [16], and even less is known regarding malignant stem cells. CSCs from dissociated spheroids exposed to 100 MOI *Fn* displayed early phosphorylation of the nuclear factor kB inhibitor IkB-α, an event heralding NF-kB activation (Figure 2A). Consistent with this finding, a factor-responsive dual-luciferase reporter assay confirmed the induction (about twofold) of NF-kB transcriptional activity in CSC after 24 h of incubation with 100 MOI *Fn* or 10 μg/mL of the bacterial wall component muramyl dipeptide (MDP) (Figure 2B)*,* while the laboratory *E. coli* strain DH5 alpha had no effect under the same experimental conditions. Of note, Western blot analysis of spheroid cell homogenates confirmed the presence of the MDP receptor and bacterial sensor NOD2 (Figure 2A). Additionally, *Fn*-treated cells displayed increased generation of nitric oxide (NO), a gaseous vasoactive and inflammatory mediator also involved in colorectal CSC identity and malignancy [29] (Figure 2C,D). Even more relevant, a 42-cytokine antibody array overlaid with CSC supernatants revealed the upregulation of two cancer-related cytokines, GRO-1 (CXCL-1) and IL-8 (CXCL-8), upon 24 h of exposure of the cell to 100 MOI of live *Fn* (Figure 2C)*,* a finding that is perfectly aligned with a recent report by Casasanta et al. [10] in HCT-116 cells. Thus, CSCs are competent for innate immune signaling and activate a proinflammatory secretory response to *Fn* conducive to a pro-carcinogenic and tumor-suppressive microenvironment.

**Figure 3:**
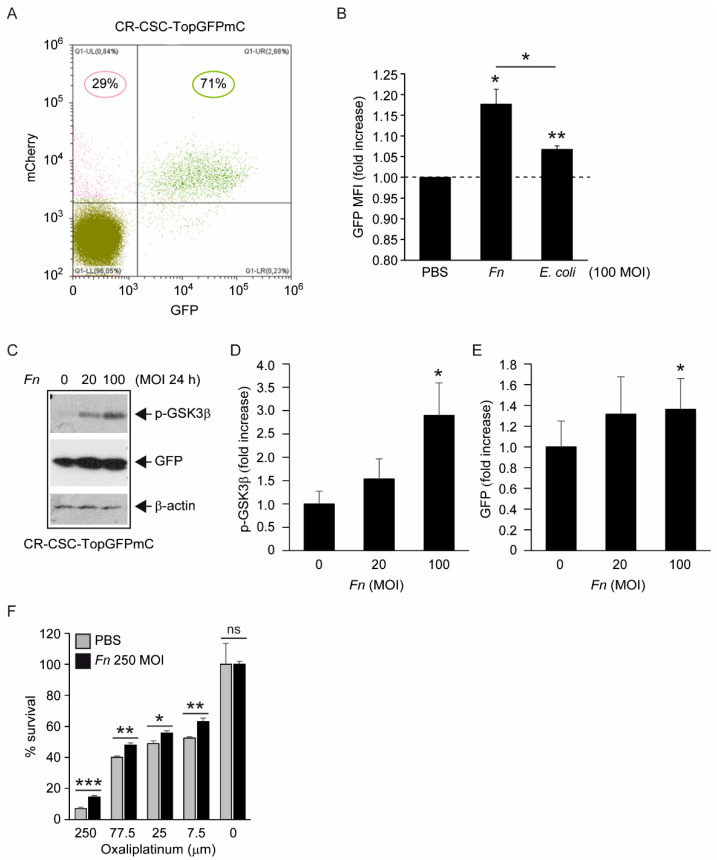
*Constitutive Wnt activity in CSC-P cells and modulation by Fn.* (**A**) Flow cytometry analysis of red/green fluorescence distribution in CSC-P cells transduced with the Wnt reporter TopGFPmC. Percentages in the upper quadrants refer to the mCherry+ population expressing the reporter. (**B**) Effect of bacterial infection on green fluorescence intensity within the GFP+/Red+ population (UR quarter). *Fn, or E. coli* were administered at 100 MOI for 48 h in a growth-factor-free medium. Values are the GFP mean fluorescence intensity (MFI) normalized to the untreated sample. Representative of two–three independent experiments; * *p* < 0.01 and ** *p* < 0.005 by a single-sample *t*-test (two-tailed). * between columns: *p* < 0.05, unpaired *t*-test. (**C**) Immunoblot detection of phospho(Ser 9) GSK3β and GFP in lysates from reporter-transduced CSC-P under the indicated treatments. Arrows highlight relevant bands and equal loading. (**D**,**E**) band densitometry from multiple (n = 3–4) experiments as in (**C**). Values are the mean ± S.E.M of the band intensities normalized to the untreated sample’s mean (*Fn* 0 MOI). * *p* < 0.05 vs. 0 MOI by ANOVA for correlated samples. (**F**) Cell-Glo^®^ luminescent survival test for the effect of heat-killed *Fn* (250 MOI) on CSC-P cell sensitivity to Oxaliplatin. Columns are the mean ± SD of triplicate samples, normalized to the mean of the untreated wells (100% survival). Raw values for the untreated samples in the PBS and *Fn* groups were 5,035,374 ± 676,612 and 4,732,458 ± 89,854 (*p* = 0.48), respectively. Picture representative of two independent experiments. * *p* < 0.05; ** *p* < 0.01; *** *p* < 0.005, unpaired Student’s *t*-test on normalized values.

Along parallel lines of investigation, the activation of Wnt/β-catenin signaling by fusobacterial FadA binding to E-Cadherin has been shown to promote CRC cell growth and survival [8]. The constitutive activation of this signaling pathway also defines colon CSCs and is pivotal for CSCs self-renewal and malignant growth in vivo [30]. To monitor signaling through the β-catenin-T cell factor/lymphoid enhancer factor (TCF/LEF) axis, CSC-P cells were transduced with a reporter construct encoding the green fluorescent protein (GFP) under the transcriptional control of a 7xTCF-responsive element [21] and exposed to bacteria (100 MOI) for 48 h in the absence of exogenous growth factors in an attempt to decrease constitutive TCF activity. As expected, CSCs displayed a high level of baseline reporter activity, with over 70% of transduced cells (marked by the PGK promoter-driven red fluorescent protein mCherry) also being GFP+ (Figure 3A). However, co-culture with *Fn* led to a modest, but detectable, increase in TCF-driven green fluorescence compared with unstimulated or *E. coli*-treated cells, as evaluated by flow cytometry (Figure 3B) and confirmed by anti-GFP immunoblotting of bulk cell homogenates (Figure 3C,E). Moreover, the induction of GFP was paralleled by a marked increase in the phosphorylation of glycogen synthase kinase (GSK)-3β on serine 9 (Figure 3C,D), an upstream event in the biochemical cascade leading to β-catenin nuclear translocation and the activation of TCF/LEF target genes [31]. Importantly, the latter response was not or marginally induced by *E. coli*, but was partially restored in this non-pathogenic strain by the recombinant expression of the fusobacterial CbpF adhesin (Supplementary Figure S4A,B). Finally, exposure to heat-killed *Fn* increased CSCs’ survival under the anticancer drug Oxaliplatin (roughly doubled % at 250 μM), while having no effect on proliferative capacity in standard medium (Figure 3F). Although not formally proved by our experiment, decreased sensitivity to chemotherapy likely reflects a Wnt-dependent response of colorectal CSC to infection [32]; thus, the above observations collectively support the conclusion that *Fn* modulates this cascade in colorectal CSCs so as to enhance their stemness and possibly promote chemoresistance and overall malignancy (see discussion below).

### 3.3. Fn Triggers CEACAM-1 Dependent Protein Tyrosine Phosphorylation Signals in Spheroidal CSCs

To further extend our mechanistic understanding of *Fn* signaling to CSCs, we focused on CEACAM-1 as an established *Fn* docking protein abundantly expressed in this malignant cell population (Figure 1F,G). While elegant studies in T lymphocytes have shown that CEACAM-1 engagement by the fusobacterial adhesin CbpF inhibits the T cell response and antitumor immunity [13,20,33], the consequences elicited by the same interaction on the tumor cell side remain largely uninvestigated. It is, however, known that CEACAM-1 exerts both positive and negative effects on CRC cell growth [34], the latter being in part mediated by the recruitment of cytosolic tyrosine phosphatases PTPN6 and 11 (formerly SHP-1 and SHP-2) through an immunoreceptor tyrosine-based inhibitory motif (ITIM) present in the 72 aa cytosolic tail of the long (L) splice variants [35]. Additionally, Src-family tyrosine kinases (STKs) can also be recruited to the CEACAM-1 intracytoplasmic domain, with the ratio between kinases and PTPases being dictated by the receptor oligomerization status [36].

**Figure 4:**
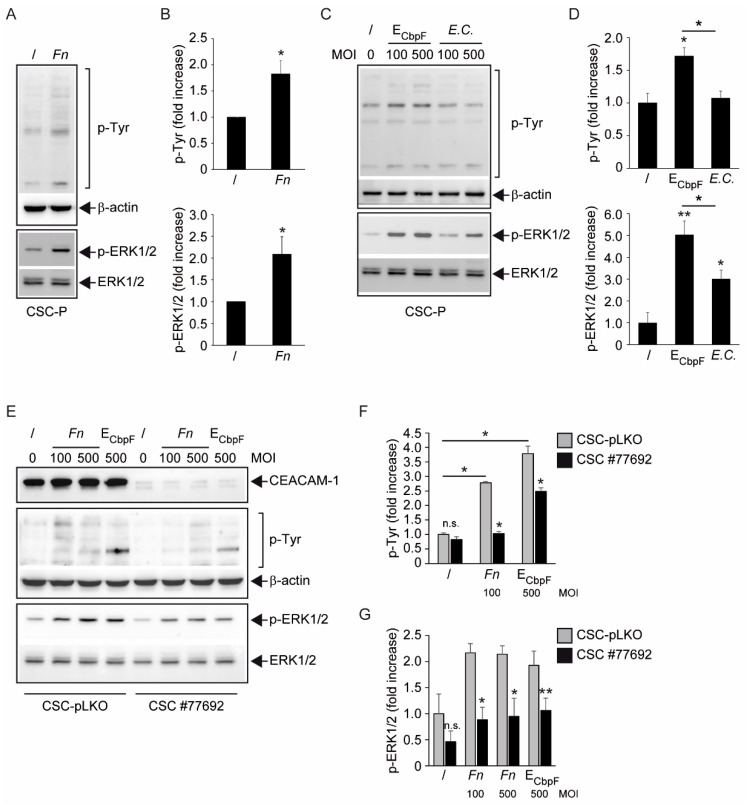
Fn *triggers CEACAM1-dependent protein tyrosine phosphorylation signals in spheroidal CSCs.* (**A**) Representative Western blot analysis of total homogenates from control (/) and *Fn*-stimulated (*Fn*) CSC-P. Cells were stimulated for 20 min with 200 MOI *Fn* in serum-free RPMI. Membranes were stained with antisera directed against tyrosine-phosphorylated proteins (upper) and phosphor-ERK1/2 (Thr202/Tyr204) (lower). Immunoblots for β-actin and total ERK1/2 are reported as a control for equal protein loading. Relevant patterns and bands are indicated by braces and arrows, respectively. (**B**) Lane (pTyr, range 60–140 kD) and band (pERK) quantitations from 7–8 independent experiments as in (**A**). Represented values are the mean fold-induction ± S.E.M. Statistics were determined by the Wilcoxon signed-rank test (* *p* < 0.02). (**C**,**D**) Representative immunoblots and relative lane/band quantitations of tyrosine-phosphorylated proteins (upper) and phospho-ERK1/2 (Thr202/Tyr204) (lower) in homogenates of CSCs treated with CbpF-expressing *E. coli* (E_CbpF_), the non-recombinant control strain (*E.C.*), or no bacteria (/). MOI (100 or 500) are indicated. Loading controls are as in (**A**). Values are the mean ± S.E.M. of band intensities from multiple (range 2 to 6) experiments, normalized to the mean of the untreated samples. Statistics (* *p* < 0.05; ** *p* < 0.01 vs. untreated; black line = EcbpF vs. *E.C.*) were determined by ANOVA (Tukey HSD post hoc test). (**E**) Immunoblot analysis of protein phosphorylation signals in CSC cells depleted of CEACAM-1 (#77692) and their mock-infected (pLKO) controls. Cells were stimulated as in (**A**) with the indicated MOI of *Fn* or E_CbpF_. Stainings and loading controls as in A and C. Successful silencing of CEACAM1 in #77692 cells was verified by anti-CEACAM1 immunoblotting (top panel). (**F**,**G**) Lane/band densitometry from multiple (2 to 3) experiments. Values are the mean ± S.E.M of the band intensities, normalized across the treatments to the mean of the pLKO (/) sample. Statistics were performed on non-normalized values by either two-way ANOVA (panel (**F**), * *p* < 0.01 (#77692 vs. pLKO; pLKO treated vs. untreated), or a paired Student’s *t*-test (panel (**G**), ** *p* < 0.005 vs. pLKO; * *p* < 0.05 vs. pLKO). n.s. denotes non-significance.

CSCs from dissociated spheroids exposed to *Fn* in the absence of exogenous growth factors displayed a rapid (20 min) increase in tyrosine-phosphorylated protein species, as revealed by anti-phosphotyrosine immunoblotting (Figure 4A,B, upper panels). This growth-factor-like signaling activity was paralleled by phosphorylation at Thr 202-Tyr 204 of the p44/42 mitogen-activated protein kinase (MAPK, ERK1/2), a crucial transducer downstream of protein tyrosine kinase (PTK) receptors (Figure 4A,B, lower panels). Importantly, the same CbpF-expressing *E. coli* strain previously shown to precipitate CEACAM-1 from CSC homogenates (Figure 1H) also elicited protein tyrosine phosphorylation and ERK1/2 activation in spheroid cells nearly as efficiently as *Fn* (Figure 4C,D). To confirm the involvement of CEACAM-1 in these biochemical responses, we stably inhibited its expression in CSC-P cells by the lentiviral transduction of a commercial shRNA construct, followed by selection in puromycin. Western blot analysis (>80% reduction of band intensity) and flow cytometry confirmed a substantial decrease in the total and surface CEACAM-1 expression, respectively, in shRNA-transduced cells (henceforth CSC-#77692) compared with mock-infected controls (CSC-pLKO) (Figure 4E and Supplementary Figure S5).

CEACAM-1 knock-down did not result in prominent cell phenotypic changes, although a slight increase in cell proliferative rate was observed (Supplementary Figure S5D). However, commensurate to the reduced expression of CEACAM-1, both total protein tyrosine phosphorylation and ERK1/2 activation in response to 100–500 MOI *Fn* were markedly attenuated in CSC-#77692 compared with pLKO control cells (Figure 4E–G). Likewise, CEACAM-1 knock-down diminished ERK1/2 activation by CbpF-expressing *E. coli* and reduced the bacterial induction of several phosphoprotein bands in the 60–180 kD range (Figure 4E–G). Collectively, these data confirm that the growth factor-like phosphorylation cascade triggered by *Fn* in colorectal CSCs is, at least in part, mediated by CEACAM-1 and recapitulated by the fusobacterial adhesin CbpF1 expressed in *E. coli*.

### 3.4. Fn Modulates CEACAM-1 Interaction with the Non-Receptor PTPase SHP-2 in CSCs

**Figure 5:**
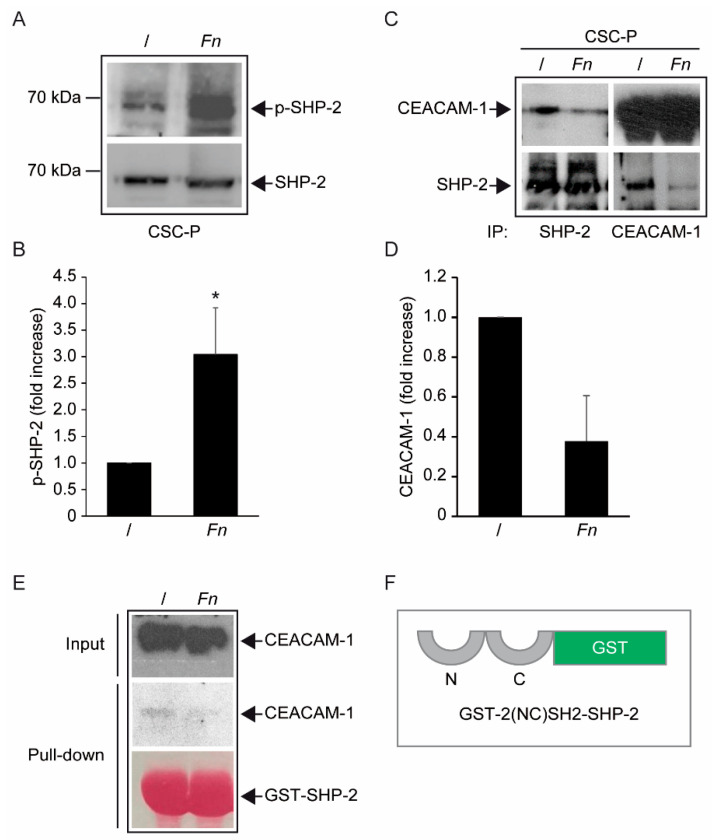
*Effect of Fn on the CEACAM1-SHP2 complex in spheroidal cells.* (**A**) Immunoblot detection of phospho-SHP-2 (Tyr542) and total SHP-2 in total cell lysates from *Fn*-treated (100 MOI for 20 min) CSC-P cells. Relevant bands around 70 kD are indicated by arrows. (**B**) Band densitometry for p(Tyr542)SHP-2 from n = 7 independent experiments. Values are the mean ± S.E.M. of stimulation indexes (stimulated/unstimulated). Statistics were determined by the Wilcoxon signed-rank test performed on raw data.(* *p* < 0.02). (**C**) CEACAM1/SHP-2 co-immunoprecipitation assay in CSC-P cells exposed to *Fn* as in (**A**). The abundance of the two proteins in anti-SHP2 immunoprecipitates (upper and lower left quadrants) and anti-CEACAM-1 immunoprecipitates (upper and lower right quadrants) was determined by standard immunoblotting. (**D**) CEACAM-1 relative abundance in anti-SHP-2 immunoprecipitates from *Fn*-stimulated compared with untreated cells over 3 independent experiments. The error bar is the SD. (**E**) Pull-down assay on protein lysates obtained as in (**A**,**C**). A GST fusion protein encompassing the 2 (N terminal and C-terminal) SHP2 phosphotyrosine binding domains (depicted in panel (**F**)) was used as bait. CEACAM-1 in input lysates (top) and GST-SHP2 precipitates (middle) was revealed by immunoblotting. Equal amounts of immobilized bait protein across different samples were confirmed by reversible Ponceau-S staining (bottom panel).

Since *Fn* modulates protein tyrosine phosphorylation through CEACAM-1, we reasoned that bacterial engagement could alter the localization and/or activity of the phosphatases associated with this ITIM-containing adhesion molecule. Western blot analysis of the total cell homogenates confirmed that both SHP-2 (PTPN11) and SHP-1 (PTPN6) were abundantly expressed in CSCs (Figure 5A; Supplementary Figure S7). Interestingly, immunoblotting with a phospho-specific reagent revealed that SHP-2 undergoes phosphorylation at Tyr 542 upon cell exposure to *Fn*, consistent with the involvement of this enzyme in the cell’s response to the pathogen (Figure 5A,B). As expected, CEACAM-1 and SHP-2 could be reciprocally co-immunoprecipitated, although with low stoichiometry, as a stable complex in unstimulated CSCs (Figure 5C). To our surprise, *Fn* markedly reduced such interaction (Figure 5C,D). Importantly, *Fn* did not appreciably change the amount of precipitable CEACAM-1 and SHP-2 in the input homogenates (Figure 5A,C,E and Supplementary Figure S7), suggesting that a reduced protein–protein interaction, rather than complex degradation or partition to an insoluble cell fraction, accounts for the decreased co-immunoprecipitation signal. In keeping with this interpretation, *Fn* treatment also diminished the binding of CEACAM-1 to a recombinant GST fusion protein encoding the two SHP2-SH2 phosphotyrosine binding domains, used as a bait in a pull-down assay (Figure 5E,F). However, although *Fn*-dependent dephosphorylation of CEACAM-1 intracytoplasmic ITIM remains the most likely explanation for this finding, we could not detect CEACAM-1 tyrosine phosphorylation, irrespective of the stimulation with *Fn* (Supplementary Figure S8).

Thus, while these observations point to CEACAM-1/SHP-2 as a novel, potentially actionable signaling axis downstream of *Fn*’s interaction with colorectal CSCs, mechanistic details deserve further clarification.

## 4. Discussion

Intestinal bacteria have recently drawn unprecedented attention as a determinant of human health and disease; this is especially true for inflammatory bowel diseases and colorectal cancer as their most severe and worrisome complications. The proposed model whereby altered bacterial–host communication and the ensuing inflammatory response leads to un-resolving epithelial damage–regeneration cycles, eventually conducive to malignant transformation and carcinogenesis, implies a primary involvement of intestinal stem cells in the process; however, our understanding of whether and how stem cells, and particularly CSCs, communicate with the microbiota is still incomplete [16]. The present work preliminarily addresses this critical knowledge gap by investigating the interaction between CRC stem-like cells and *Fn*, a pathobiont recently recognized as a potential etiologic factor in colon cancer development and malignant progression. Our results provide evidence for a direct interaction of bacteria with immature cancer cells, investigate the downstream cellular responses, and highlight a novel signaling circuitry whereby *Fn* triggers a growth-factor-like signaling cascade in CSC by impinging on the complex between the bacterial receptor CEACAM-1 and its associated cytosolic tyrosine phosphatase SHP-2.

One first element of novelty in the research presented here is the focus on a stem-like cancer cell population. Although spheroidal cultures of colon cancer cells can still be heterogeneous in terms of clonogenicity and malignant potential, evidence for the (a) expression of CD133 in the majority of CSC-P cells, (b) downregulation of the pluripotency factors Nanog and Oct4 in differentiative culture conditions, and (c) constitutive activation of the Wnt pathway as revealed by the fluorescent reporter TopGFP clearly argues in favor of this cell model being representative of the CRC stem cell subset. Additionally, experiments of cell–bacteria interactions with fluorescent *Fn* confirm that nearly 100% of spheroid-derived cells are physically targeted by the pathogen. Therefore, it is unlikely that the cell responses to *Fn* infection described here are accounted for by a minority of more differentiated cells phenotypically similar to cancer cell lines employed in previous studies.

The finding of the direct and stable binding of *Fn* to CSC, presented in Figure 1, is not trivial. The reported enrichment of *Fn*, not only at primary CRC sites, but also in distant metastatic colonies [4], may, in fact, reflect the capacity of the pathogen to hitchhike a specific subpopulation of tumor-initiating and tumor-disseminating cells. On the other hand, while CSCs appear to express multiple potential *Fn* docking molecules (Gal-GalNac, E-cadherin, CEACAM-1), they may not be easily accessible for microbial contact when confined to their hypoxic niche in vivo. Nevertheless, spontaneous or therapy-induced tumor necrosis may occasionally expose CSCs so as to infringe their isolation. Additionally, Gal-GalNac expression (Figure 1E) renders CSCs targetable by systemically spread *Fn*, and even more so in hypoxic regions, which is more favorable for this obligate anaerobic pathogen. Notably, the spheroid 3D culture model creates a unique microenvironment permissive to the growth of anaerobic bacteria [37], which adds to the relevance of this experimental setting for studying the interaction between *Fn* and CSCs.

The molecular analyses displayed in Figure 2 clearly show that *Fn* can elicit proinflammatory and innate immune responses in intestinal cells, including the activation of NF-kB and secretion of the cancer-associated chemokines CXCL1 (Gro-α) and CXCL-8 (IL-8) [10]; while in part confirmative, these observations gain particular relevance in revealing the potential of CSC to actively participate in the establishment of a tumor-promoting and immunosuppressive microenvironment. From this perspective, of special interest is the *Fn*-elicited generation of nitric oxide, a gaseous mediator involved in microbicidal responses in macrophages, but also previously linked to colorectal CSCs’ malignant capacity [29]. Along a parallel line of speculation, while our data show that spheroid-derived cells are competent for bacterial sensing, it is still possible that the antibacterial responses triggered in these progenitors are somewhat less efficient than those in mature epithelial cells, making them a preferential infection target for *Fn* or other pathogens. This possibility, which entails far-reaching implications for microbe-driven colorectal carcinogenesis, deserves further investigation by systematically comparing innate immune responses in stem versus differentiated cancer cells.

*Fn* reportedly activates Wnt signaling [8] and produces CSC characteristics and a mesenchymal phenotype in CRC cells [17,18]. The results depicted in Figure 3 complement this information, showing that, although constitutively active, the Wnt cascade can be further stimulated by *Fn* in CSC, as revealed by the increased inhibitory phosphorylation of the β-catenin destruction factor GSK3β [31] and by the induction of TCF/LEF-driven recombinant GFP. Additionally, the increased chemoresistance of CSCs to oxaliplatin when co-cultured with *Fn,* although not directly demonstrated in the present work, is consistent with enhanced stem-like features downstream of Wnt activation. Of note, Vermeulen et al. elegantly showed that Wnt signaling in CSCs can be triggered by extrinsic cues, such as HGF secreted by stromal myofibroblasts [30]. Thus, by adding *Fn* to the list of “environmental” stemness-promoting factors, our observations align perfectly with this idea. Nevertheless, changes in cells transduced with TCF-driven GFP were relatively modest, with a slight fluorescence increase in GFP+ cells and no detectable cell shift from the GFP- to the GFP+ population (Figure 3A,B, and data not shown). Whether this reflects an exceptionally high baseline Wnt activity in our CSC line or is the result of a technical limitation (i.e., the use of non-cloned Top-GFP cells bearing varying copy numbers of the probe) is currently being evaluated.

The analysis of protein tyrosine phosphorylation events elicited by *Fn* binding to CSCs represents the most innovative contribution of the present work. This growth factor-like cascade, culminating in the phosphorylation of ERK1/2, is largely mediated by CEACAM-1, as indicated by the blunted response observed in cells depleted of this adhesion molecule (Figure 4). Accordingly, an *E. coli* strain recombinant for the fusobacterial autotransporter adhesin and CEACAM-1 ligand CbpF [11,20] elicited a similar set of cellular responses in CSCs more efficiently compared with the parental, non-CEACAM-1 binding parental EC (Figure 1H and Figure 4C,D). However, the mechanistic interactions whereby *Fn* activates an RTK-like cascade in CSCs remain largely undefined. The signaling capacity of CEACAM-1 resides in the ability of the “long” (71 aa) cytosolic tail, harbored by the L isoforms, to recruit tyrosine phosphatases (via the ITIM domain) and Src-like kinases [38]. Notably, the relative affinity toward these different transducers is regulated by homophilic interactions and receptor cis-dimerization dictated by the extracellular domains. Rigorous FRET-based studies by Muller et al. suggested that transmembrane signaling by CEACAM-1 operates by altering the monomer/dimer equilibrium, which leads to changes in the SHP-2/c-Src–binding ratio [36]. Thus, *Fn*, by engaging CEACAM-1 through CbpF, could impact the supramolecular organization of CEACAM-1-L aggregates so as to tilt the phosphorylation–dephosphorylation balance in favor of the former. In keeping with this view, the co-immunoprecipitation studies presented in Figure 5 are consistent with the dissociation of the CEACAM-1–SHP2 complex in response to bacterial stimulation, although the possibility of a simultaneous recruitment and the activation of Src-like kinases by CEACAM-1 was not directly investigated. Notably, the above model diverges from the one proposed by Bachrach and colleagues for the immunosuppressive action of *Fn*-CEACAM-1 signaling in T lymphocytes and NK cells, whereby bacterial engagement leads to CEACAM-1 dimerization/activation, and possibly the phosphatase-dependent downregulation of antigen-triggered activation signals [20,33].

The mechanism leading to SHP-2 dissociation from CEACAM-1 upon *Fn* binding remains elusive, although the dephosphorylation of the CEACAM-1 ITIM at positions Y493 and Y520, the putative SHP-2 docking sites, represents a plausible explanation. This idea is consistent with the reduced CEACAM-1 recovery from *Fn-*stimulated CSC lysates in pull-down experiments, where the two SHP-2 SH2 phosphotyrosine binding domains were used as bait (Figure 5E). Unfortunately, we could not detect constitutive CEACAM-1 tyrosine phosphorylation in unstimulated CSCs, nor dephosphorylation upon *Fn* challenge (Supplementary Figure S8); future experiments with CSCs expressing CEACAM-1 mutant forms lacking the ITIM region will hopefully help to clarify this point.

In a broader perspective, our results carry significant implications for CRC biology. While recent works have focused on the immunomodulatory and immunosuppressive consequences of CEACAM-1 engagement by *Fn* in T and NK cells, the data presented here underscore the relevance of this pathogenic interaction on the cancer cell side. Relevant to this aspect, CEACAM-1 has been recognized as playing a dual role in colorectal cancerogenesis, possibly tumor-suppressive in the early phases, and supportive for malignant progression and metastasis in advanced disease [39,40]. The evidence presented for *Fn* dissociating CEACAM-1 from its inhibitory effector SHP-2 is consistent with the well-established paradigm whereby microbial carcinogenesis targets tumor suppressor mechanisms [41]. On the other hand, by operating in CSCs, the *Fn*–CEACAM-1–SHP2 axis qualifies well to link bacterial infection with cancer progression and dissemination. In keeping with this attractive hypothesis, CEACAM-1 is highly expressed in EpCAM+ liver CSCs [42], and its overexpression induces stem cell markers and EMT, a stem-cell-related phenomenon, in HT29 and HCT16 CRC cell lines [43]. Additionally, CD66c (a.k.a. CEACAM-6), another member of the CEACAM family, has been reported to mark a population of CD133+ CR-CSC, and its downregulation arrests tumor growth in vivo [44]. While the preliminary studies presented in Supplementary Figure S9 suggest that CEACAM-1 protein expression is not affected by CSC differentiation, we are currently evaluating whether the CEACAM-1 signaling properties, and by extension biological responses to *Fn*, vary across spheroidal cell subsets in a fashion that correlates with stemness and Wnt signaling capacity. Likewise, further studies aimed at better dissecting the role of the CEACAM-1–SHP axis in the proinflammatory and Wnt-related responses elicited by *Fn* in CSCs (Figure 2 and Figure 3) are warranted [45,46].

## 5. Conclusions

In conclusion, the present work provides evidence for a direct interaction between *Fn*, a pathogen involved in colorectal carcinogenesis, and tumor spheroid cultures highly enriched in colon CSCs. Furthermore, our data not only confirm, in CSCs, previously described proinflammatory and protumorigenic activities of *Fn*, but also identify a signaling axis involving CEACAM-1, SHP-2, and the downstream tyrosine phosphorylation cascade as potentially relevant for CSCs’ biological response to the pathogen. Thus, although in part preliminary, these observations entail broad implications for microbial carcinogenesis in the intestine and outline novel actionable mechanisms for preventive and therapeutic interventions. Additionally, by revealing *Fn*’s capacity to bind CSCs, our findings support the idea of exploiting this tumor-associated microbe as an engineerable platform to target CRC and other common malignancies [47].

## Data Availability

Not applicable.

## Supplementary Materials

The following supporting information can be downloaded at: https://www.mdpi.com/article/10.3390/biom12091256/s1. Figure S1: Persistent bacteria-associated fluorescence and bacterial DNA in CSC-P cells infected with green-labeled *Fn*. Figure S2: Lack of E-cadherin bactoprecipitation by *Fn*. Figure S3: Forward-scatter gating of live CSCs in flow cytometry studies. Figure S4: Phosphorylation of GSK3β in response to *Fn*, but not *E. coli*, in CSC-pLKO cells. Figure S5: Increased proliferative capacity of CEACAM-1-depleted cells compared with non-targeted controls. Figure S6: RayBio^®^ C-SeriesHuman Cytokine Antibody Array C3 Map. Figure S7: SHP-2 and SHP-1 expression in CSCs. Figure S8: No evidence for CEACAM-1 phosphorylation on tyrosine in *Fn*-stimulated CSC-P cells. Figure S9: CEACAM-1 expression level is not modulated by CSC differentiation.
