# Supplementary material for: Proinflammatory and Cancer-Promoting Pathobiont Fusobacterium nucleatum Directly Targets Colorectal Cancer Stem Cells
Source: Biomolecules. 2022 Sep 7;12(9):1256. doi: 10.3390/biom12091256 (PMC9496236; doi:10.3390/biom12091256)
Supplement: Supplementary file 1 [file biomolecules-12-01256-s001.zip › biomolecules-1837272-supplementary.pdf]

biomolecules

MDPI

Article

Proinflammatory and cancer-promoting pathobiont *Fusobacterium nucleatum* directly targets Colorectal Cancer Stem Cells

Virve Cavallucci, Ivana Palucci, Marco Fidaleo, Antonella Mercuri, Letizia Masi, Valeria Emoli, Giada Bianchetti[6], Micol Eleonora Fiori, Gilad Bachrach, Franco Scaldaferri, Giuseppe Maulucci, Giovanni Delogu, Giovambattista Pani

Supplementary Figures

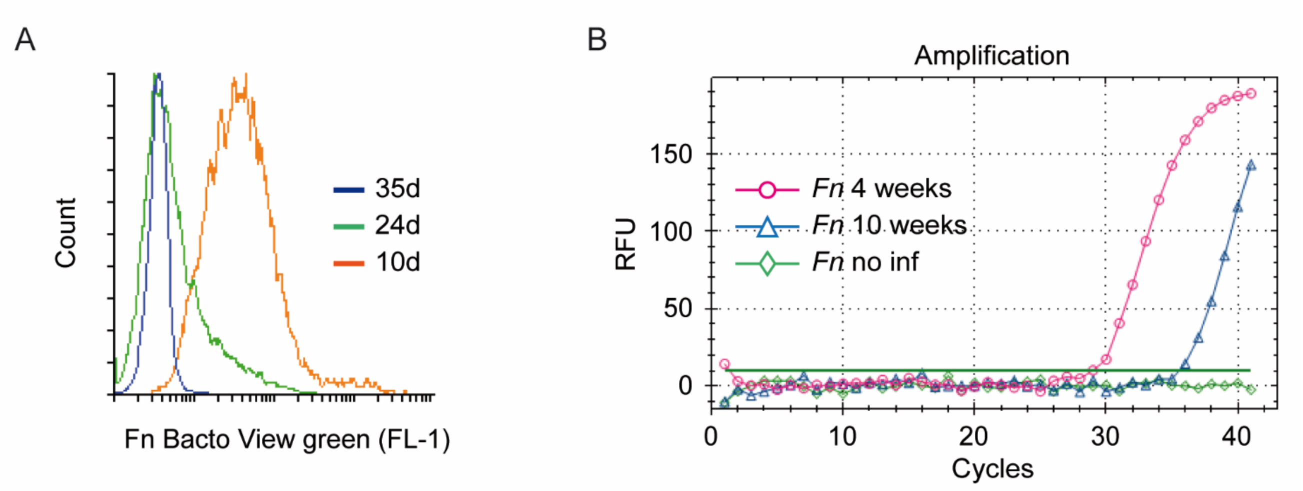

Figure S1. *Persistent bacteria-associated fluorescence and bacterial DNA in CSC-P cells infected with green-labelled Fn.* A. Flow cytometry single parameter histogram overlay displaying CSC green fluorescence at the indicated days after infection with 100 MOI of BactoView™ Live Green-labelled *Fn.* The percentage of fluorescent cells was near 100%, 45% and <1% at 10, 25 and 35 days, respectively. B. Real time qPCR plot illustrating the detection of *Fn* DNA of cells as in A. Threshold cycle (Ct) was 28 at 4 weeks and 36 at 10 weeks. Lack of DNA amplification in non-infected cells confirms reaction specificity.

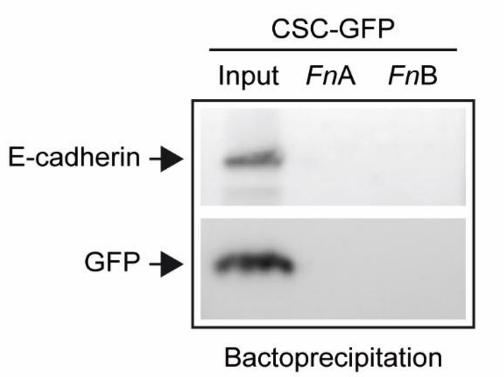

Figure S2. *Lack of E-cadherin bactoprecipitation of by Fn.* Protein lysates from CSC-GFP cells were incubated with two different preparations of Fn (A and B, same strain 25686). The presence of the protein band in the input lysate, but non in bacterial precipitate is highlighted by the upper arrow. GFP was used as a control for binding specificity as in figure 1H).

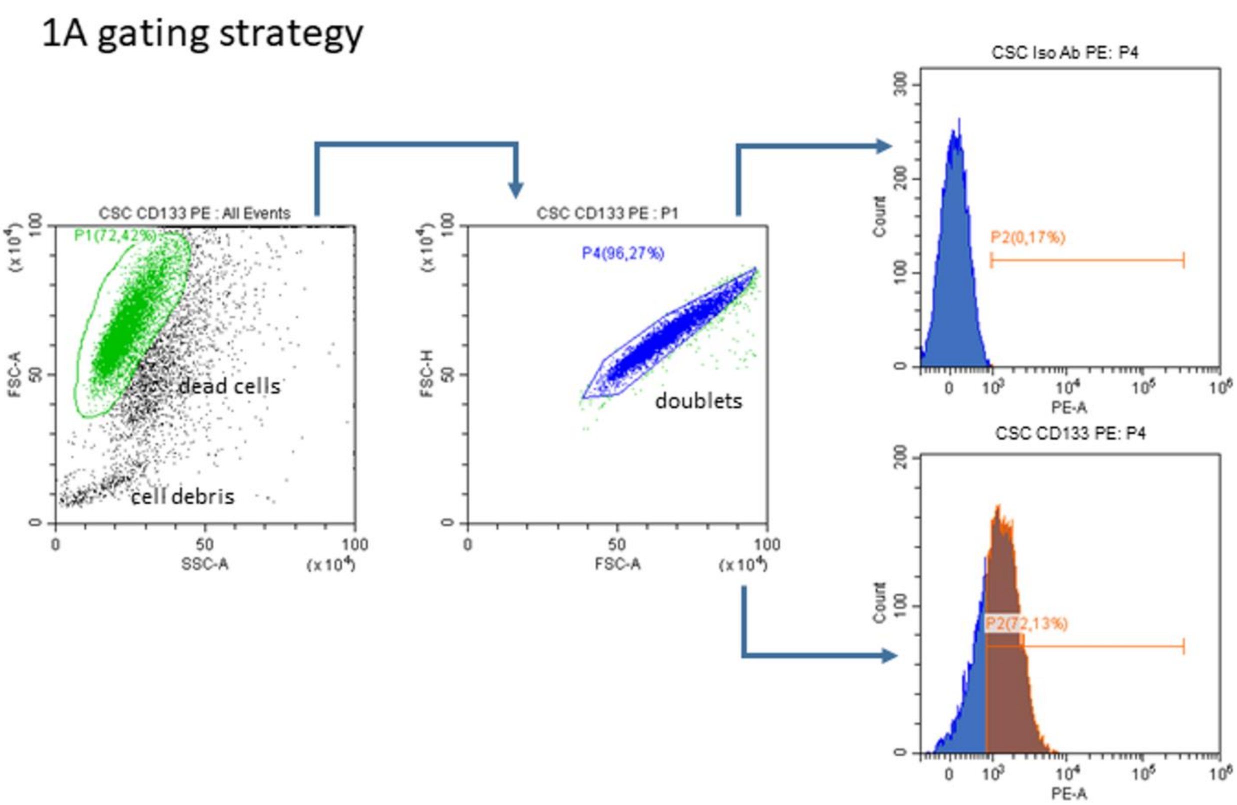

Figure S3. *Gating strategy for live single CSCs in flow cytometry studies (refers to figure 1A).* Dead cells (low FSC, high SSC) and debris (low FSC, low SSC) were excluded from the Forward/Side scatter plot. Cell doublets/clusters were removed from the target population (P1) as events with high FSC Area/Hight ratio. The resulting population (P4) was analysed for marker expression or unspecific staining. Propidium Iodide staining further confirmed the identification of excluded events as dead cells/debris.

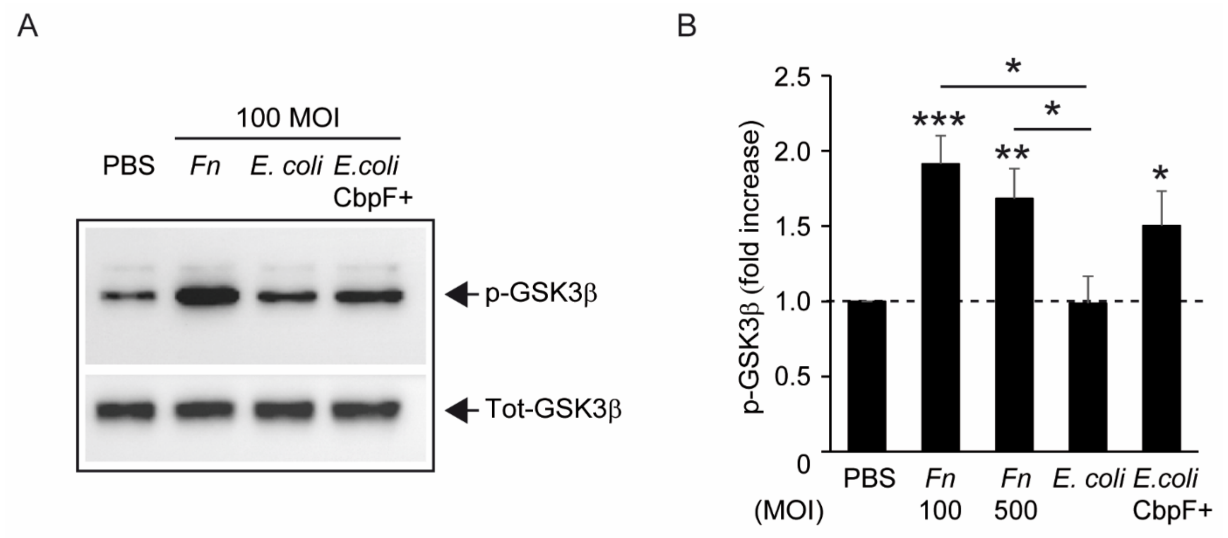

Figure S4. *Phosphorylation of GSK3b in response to Fn but not E. coli in CSC-pLKO cells.* A. Representative blot displaying GSK3β phosphorylation in CSC-pLKO cells exposed for 24 hours to *Fn* (100 or 500 MOI) *E. coli* (500 MOI)or recombinant CbpF+ *E.C.* (500 MOI). Cells were treated as in figure 3B, except that all bacterial strains used in this set of experiments were heat-killed to prevent overgrowth in culture. B. Bar chart reporting mean±s.e.m. of multiple experiments (n=3-6); values are expressed as fold induction

over non-stimulated cells. ****p<0.005; **p<0.02; *p<0.05;* statistics by single sample two-tailed t-test. * between columns: *p*<0.05, unpaired *t*-test (two-tailed).

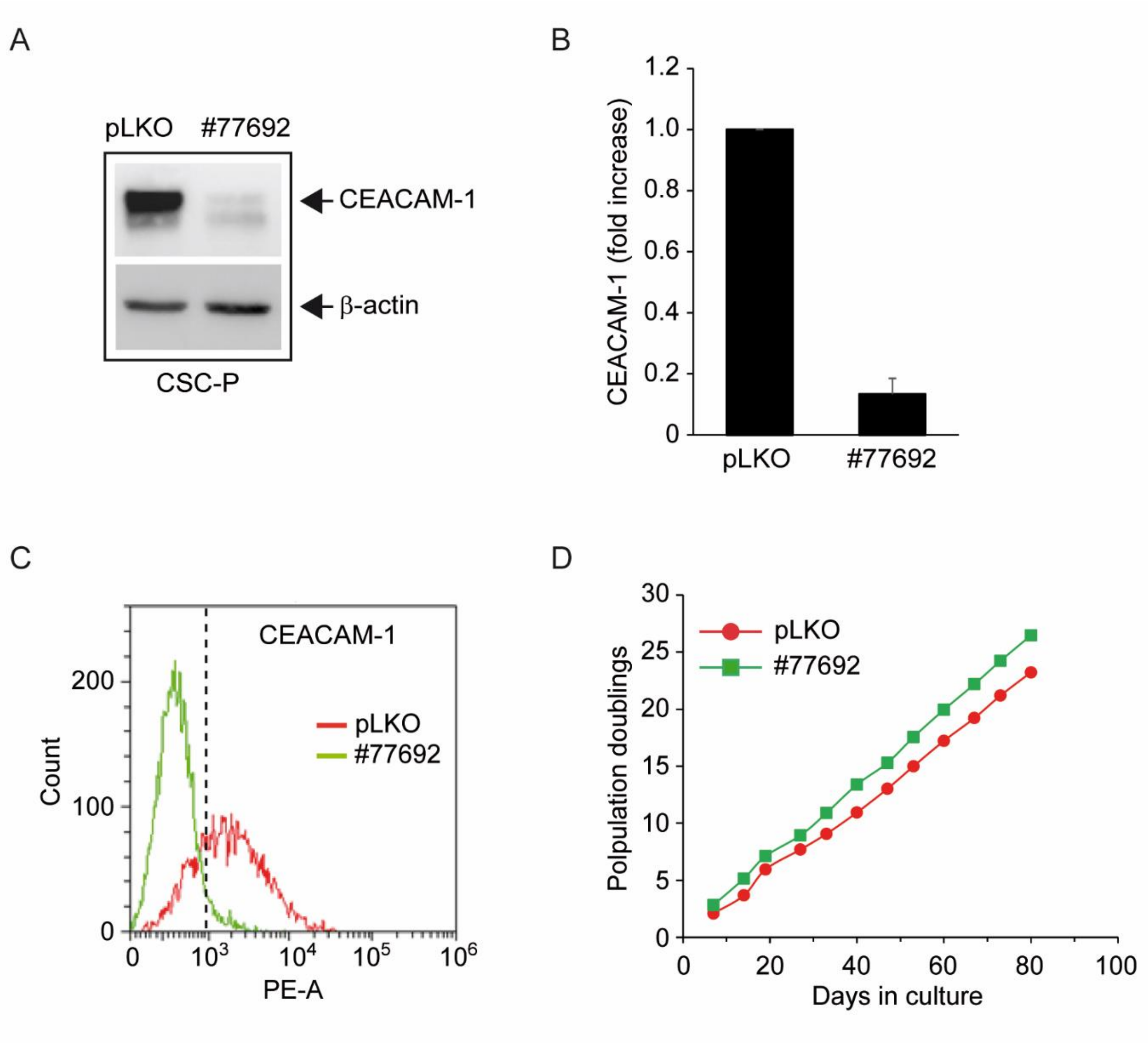

Figure S5. *Increased proliferative capacity of CEACAM-1-depleted cells compared to non-targeted controls*. A, C. Representative western blot analysis and flow cytometry single parameter histogram confirming robust downregulation of CEACAM-1 in CSC-#77692 compared to CSC-pLKO cells. B band quantitation from three independent blots. Values are mean ± SD % of CSC-pLKO. D. Curves represent cumulative population doublings (PD) over the indicated time. Cells were dissociated and counted weekly by a hemocytometer. At each passage, 10^6 cells were re-seeded in 25 cm^2 ultra-low attachment culture flasks containing 1/3 conditioned medium, 2/3 fresh medium and 3 µg/ml puromycin. PDs were calculated as

$PD = \log_2 \left[\frac{N_t}{N_0} \right]$Nt (n of cells at time t) N0 (number of cells at time 0).

Each antibody is spotted in duplicate vertically

	A	B	C	D	E	F	G	H	I	J	K	L
1, 2	POS	POS	NEG	NEG	ENA-78 (CXCL5)	GCSF	GM-CSF	GRO a/b/g	GROα (CXCL1)	I-309 (TCA-3/CCL1)	IL-1α (IL-1 F1)	IL-1β (IL-1 F2)
3, 4	IL-2	IL-3	IL-4	IL-5	IL-6	IL-7	IL-8 (CXCL8)	IL-10	IL-12 p40/p70	IL-13	IL-15	IFNγ
5, 6	MCP-1 (CCL2)	MCP-2 (CCL8)	MCP-3 (CCL7)	M-CSF	MDC (CCL22)	MIG (CXCL9)	MIP-1δ	RANTES (CCL5)	SCF	SDF-1	TARC (CCL17)	TGFβ1
7, 8	TGFα	TGFβ	EGF	IGF-1	Angio-genin	OSM	TPO	VEGF-A	PDGF-BB	Leptin	NEG	POS

Figure S6. *RayBio® C-SeriesHuman Cytokine Antibody Array C3 Map*. Duplicate spots corresponding to cytokines discussed in the text are highlighted.

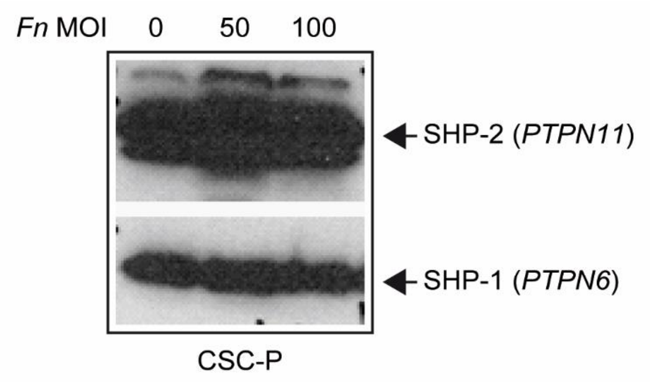

Figure S7. *SHP-2 and SHP-1 expression in CSCs*. Immunoblot analysis of CSC-P total homogenates displaying strong immunoreactivity for both SHP-2 and SHP-1 PTPase. Relevant bands are indicated by arrowheads. Note that total PTPase content is not or marginally affected by acute cell exposure to Fn.

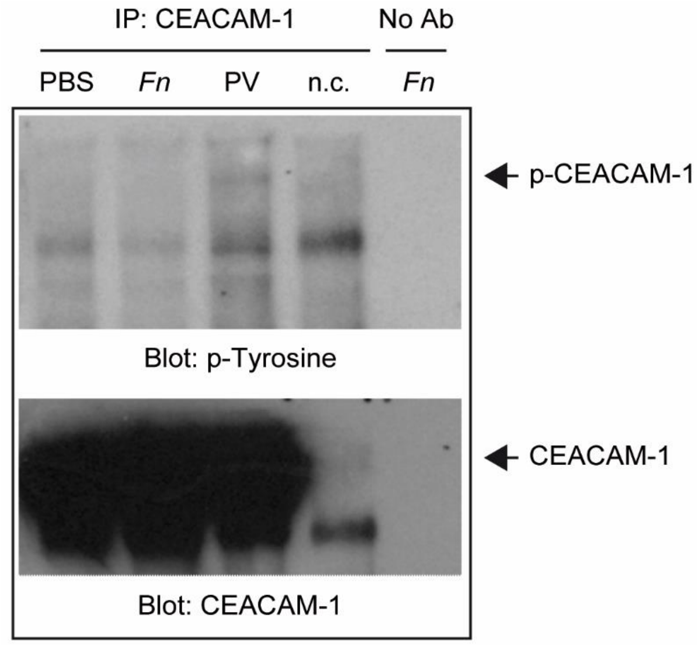

Figure S8. *No evidence for CEACAM-1 phosphorylation on tyrosine in Fn-stimulated CSC-P cells.* anti CEACAM-1 immunoprecipitates obtained as in figure 5C were immunoblotted with the antiphosphotyrosine monoclonal Ab 4G10. Cells treated with the potent PTPase inhibitor Pervanadate (PV) were used as positive control. A weak antiphosphotyrosine-reactive band of the expected size, present in the PV-treated sample is indicated by the arrowhead.

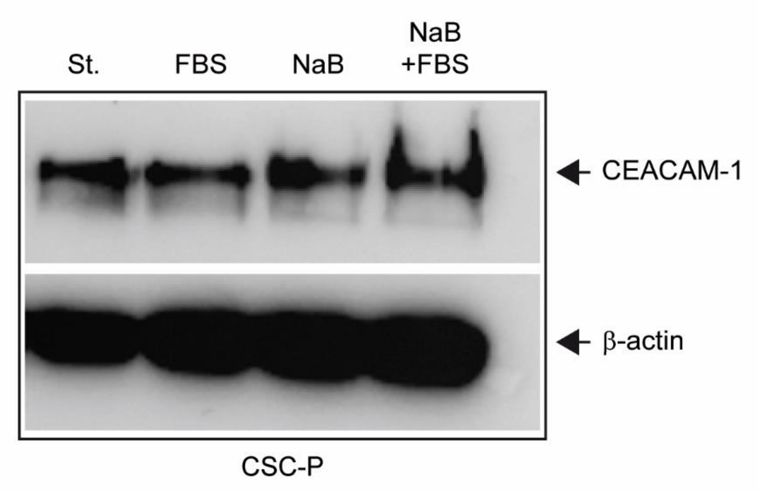

Figure S9. *CEACAM-1 expression level is not modulated by CSC differentiation.* CSC-P cells were cultivated in standard medium (St.), or in St. medium containing 2% FBS, Sodium Butyrate 10 mM (NaB) or their combination for ten days. Equal amounts of total cell homogenates were immunoblotted for anti CEACAM-1, or actin-β as a loading control.
